# The Nrf-2/HO-1 Signaling Axis: A Ray of Hope in Cardiovascular Diseases

**DOI:** 10.1155/2020/5695723

**Published:** 2020-01-30

**Authors:** Xueyan Zhang, Yihan Yu, Hanyu Lei, Yufeng Cai, Jie Shen, Ping Zhu, Qingnan He, Mingyi Zhao

**Affiliations:** ^1^Department of Pediatrics, The Third Xiangya Hospital, Central South University, Hunan Province, Changsha 410013, China; ^2^Xiangya School of Medicine, Central South University, Hunan Province, Changsha 410013, China; ^3^Xiangya School of Life Science, Central South University, Hunan Province, Changsha 410013, China; ^4^Guangdong Cardiovascular Institute, Guangdong Provincial People's Hospital, Guangdong Academy of Medical Sciences, Guangzhou, Guangdong 510100, China

## Abstract

Cardiovascular disease, which can lead to angina and shortness of breath, remains one of the most serious threats to human health. Owing to its imperceptible symptoms, it is difficult to determine the pathogenesis and treatment methods for cardiovascular disease. Nuclear factor erythropoietin-2-related factor 2/heme oxygenase 1 (Nrf2/HO-1) is a protein found in all cells of the human body. It is activated, transferred to the nucleus, and bound to DNA by antioxidant response elements (AREs). As a regulator of the antioxidant system, it upregulates the expression of HO-1 to reduce oxidative stress. Nrf2/HO-1 also has the ability to modulate calcium levels to prevent ferroptosis, pyroptosis, autophagy, programmed cell necrosis, alkaliptosis, and clockophagy. In view of the importance of Nrf2/HO-1 in the regulation of homeostasis, this review summarizes current research on the relationship between cardiovascular disease and Nrf2/HO-1. Normal cardiovascular diseases, such as viral myocarditis and myocardial ischemia-reperfusion injury, have been treated with Nrf2/HO-1. Rheumatic heart disease, cardiac tumors, arteriosclerosis, arrhythmia, hypertensive heart disease, and myocardial infarction have also been treated during experiments. Research has demonstrated the clinical application of Nrf2/HO-1 in pediatric cardiovascular disease; further clinical trials will help elucidate the potential of the Nrf2/HO-1 signaling axis.

## 1. Introduction

Cardiovascular disease is the most serious threat to the health and quality of life of humans. In 2016, an estimated 17.9 million people died of cardiovascular disease, accounting for 31 percent of all deaths worldwide. Currently, the World Health Organization (WHO) Cardiovascular Disease Program works to prevent, manage, and monitor cardiovascular disease globally. Identifying potential functional mechanisms and effective therapeutic drugs to reduce the incidence, prevalence, and mortality of cardiovascular disease has become a universal concern. One approach to preventing pathophysiological and biochemical damage is to use the body's own self-defense mechanism. The autonomic nervous system (ANS), formerly known as the vegetative nervous system, is a controlling system that largely acts unconsciously and regulates bodily functions. It plays an important role in maintaining and regulating homeostasis, indicating that dysfunction of the ANS caused by oxidative stress induces inflammation and exaggerates oxidative stress. Several cytokines triggered by the ANS are involved in the process of self-defense. When nuclear factor erythropoietin-2-related factor 2 (Nrf2) is activated in the nucleus, it turns on the production of antioxidant enzymes such as catalase, glutathione (GSH), and superoxide dismutase (SOD). These antioxidant enzymes neutralize up to one million free radicals per second. This is an effective approach for reducing oxidative stress, inflammatory response, necrosis, apoptosis, ferroptosis, alkaliptosis, and clockophagy.

The presence of three branches—the sympathetic nervous system, the parasympathetic nervous system, and the enteric nervous system, which constitute the ANS—inside the human body helps trigger vital pathways, such as the Nrf2/HO-1 pathway, when an organism suffers from oxidative stress. The oxidative stress secondarily triggers the synapses. Inhibitory and excitatory synapses between neurons control the internal system by releasing cytokines, which may lead to the activation of relevant pathways. On activation of the Nrf2/HO-1 pathway, the ANS is gradually modified to adapt to the following organ process. Through inflammation and oxidative stress, the ANS helps restore homeostasis. Hence, the effects of the ANS are associated with the Nrf2/HO-1 pathway.

**Figure 1:**
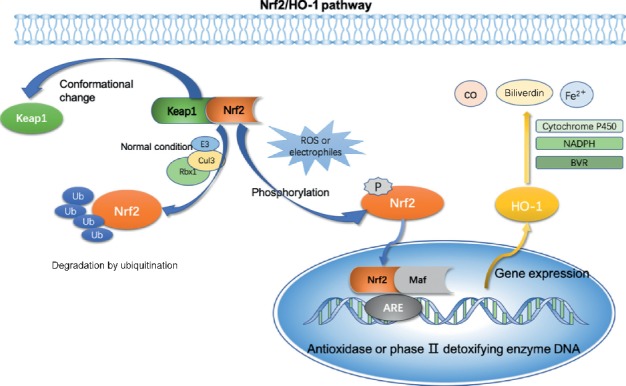
Specific signal transduction of the Nrf2/HO-1 signaling axis.

Nrf2 is a critical redox-sensitive transcription factor. It is activated to improve the oxidative stress state of the body, promote cell survival, and maintain the redox homeostasis of cells by regulating the induced expression of phase-II detoxifying enzymes and antioxidant enzymes [1]. Nrf2 protein is expressed in various tissues of the body (such as liver, kidney, spleen, and heart), and contains seven structural domains (Neh1–Neh7). Kelch-like ECH-associated protein-1 (Keap1) has two characteristic domains, namely, the dimerized domain of broad complex-tramtrack-bric-a-brac (BTB) and the double glycine repeat (DGR). The association between Nrf2 and Keap1 is realized through its N-terminal Neh2 domain, which interacts with DGR and negatively regulates Nrf2 function. When cells are attacked by reactive oxygen species (ROS) or electrophiles, Nrf2 dissociates from Keap1 and is rapidly transferred to the nucleus. Phosphorylated Nrf2 forms a heterodimer with Maf protein and then combines with antioxidant response elements (AREs), which activate the expression of heme oxygenase 1 (HO-1) [2]. In addition, various protein kinases, such as mitogen activated protein kinases (MAPKs), protein kinase C (PKC), and (phosphoinositide 3-kinase (PI3K), participate in the regulation of Nrf2 transcriptional activity by inducing phosphorylation of Nrf2. The specific signal transduction is depicted in Figure 1.

HO-1 is an important endogenous antioxidant and constitutes an important defense system. Activated by Nrf2, HO-1, and its metabolites, including CO, Fe^2+^, and biliverdin, can prevent excessive oxidation of lipids and proteins by scavenging hydroxyl-free radicals, singlet oxygen, and superoxide anions, and play an effective role in anti-inflammation, antioxidation, and anti-apoptosis [3]. Our previous studies have demonstrated that HO-1 plays a regulatory role in the induction of anti-inflammatory cytokines and adjustment in T-helper 1/T-helper 2 (Th1/Th2) and Th17/Treg ratios of immune cells [4] by activating p55/tumor necrosis factor receptor 1 (TNFR-1), p38 MAPK, and PI3K/AKT signaling axes [4, 5]. Jin et al. demonstrated that HO-1 exerts a cardioprotective effect in simulated H9C2 cells in vitro [5, 6]. Chen et al. have shown that HO-1 can reduce the amount of mitochondrial oxidation products by inducing autophagy to protect the heart [6, 7]. Available evidence indicates that HO-1 protects the systolic function of the heart and improves blood supply to target organs.

This review summarizes the important protective role of the Nrf2/HO-1 signaling axis in cardiovascular diseases. Newly discovered alkaliptosis and clockophagy not only reveal a new form of cell death but also have important implications for the prevention and treatment of biological abnormalities. Through analysis of its mechanism and specific application in disease, the Nrf2/HO-1 signaling axis provides novel insights and directions for the clinical targeted treatment and prevention of cardiovascular diseases.

## 2. Functional Regulation of the Nrf2/HO-1 Signaling Pathway

### 2.1. Nrf2/HO-1 Signaling Axis in Regulating Internal Flow of Calcium Ions

Under normal circumstances, the distribution of ions inside and outside the cell membrane is in a stable state. An excessive Ca^2+^ influx may lead to an excessive Ca^2+^ concentration in the mitochondria, Ca^2+^–dependent degradation enzyme activation, and apoptosis induction, resulting in oxidative stress and dysfunction of cells.

Jin et al. found that rutaecarpine can increase the transcriptional activity of Nrf2 and Nrf2 target genes, HO-1 and NAD(P)H quinone oxidoreductase 1 (NQO1), thereby promoting the phosphorylation of AKT and Ca^2+^/calmodulin-dependent protein kinase-II (CaMKII) [8]. Li et al. demonstrated that HgCl_2_ increases the expression of Nrf2, HO-1, and NQO1, and induces apoptosis by inducing Na^+^/Ca^2+^ overload and ATPase inactivation in mice, providing a new idea for studying the regulatory effect of Ca^2+^ ions and the molecular regulation of the Nrf2-mediated antioxidant pathway [9]. Guidarelli et al. found that inhibiting excessive Ca^2+^ influx into the mitochondria could prevent arsenite-induced cardiolipin oxidation and DNA single strand breakage, which in turn inhibits the survival signal of Nrf2 [10]. The research has demonstrated that the Nrf2/HO-1 signaling pathway is a typical antioxidant pathway and has the potential to alleviate cardiac disorders.

### 2.2. Nrf2/HO-1 Signaling Axis in Mitochondrial Oxidative Stress

During ischemia, mitochondrial electron transfer dysfunction and ROS accumulation lead to protein degradation, which then leads to mitochondrial dysfunction. The opening of mitochondrial membrane channels destroys the integrity of the mitochondrial membrane. ATP deficiency, intracellular Ca^2+^ overload, and autophagy insufficiency eventually lead to cardiac apoptosis and death.

Chen et al. showed that the potential of the mitochondrial membrane is higher than normal levels, and the mitochondrial ROS level is abruptly reduced during ischemia-reperfusion injuries [7]. Under the influence of oxidative stress, mtDNA mutations easily occur in the mitochondria, thus altering the permeability of the cell membrane [11]. Experimental studies conducted by Zhang et al. have shown that the activation of Nrf2 can reduce the degree of oxidative stress in the mitochondria and can reduce the oxidative stress and inflammatory response of vascular endothelial cells [12]. Therefore, drugs targeting oxidative stress and mitochondrial dysfunction could be used for the treatment of cardiovascular diseases in the future.

### 2.3. Nrf2/HO-1 Signaling Axis in Ferroptosis

It was previously thought that almost all regulated cell death in mammalian cells resulted from the activation of caspase-dependent apoptosis [13]. However, Stockwell et al. challenged this view by discovering regulated nonapoptotic cell death pathways. Erastin, a RAS-selective lethal compound, can trigger a unique iron-dependent nonapoptotic cell death by accumulating ROS; this type of cell death was defined as “programmed iron necrosis” [14]. Iron death is driven by the lipid peroxidation pathway, mainly through the iron metabolism and GSH depletion pathways.

Nrf2 can produce an effect on the heme-binding ferrous content by regulating intracellular heme synthesis and metabolism [15]. The most important effect is the regulation of the expression of HO-1 [16]. Nrf2 can also maintain the stability of intracellular active iron by regulating its storage and release. Human ferritin can be divided into ferritin heavy chain (FTH1) and ferritin light chain (FTL). The FTH1 gene is the target gene of Nrf2. FTH1 can oxidize Fe^2+^ to Fe^3+^ and store it. Activated Nrf2 regulates not only iron homeostasis by increasing iron storage but also the expression of the membrane iron transporter (FPN1) to regulate iron ions in and out of cells. FPN1 is the only protein that can transfer iron ions out of cells. Recent studies have also shown that Nrf2 activation can upregulate the expression of genes involved in iron metabolism and ROS metabolism.

By injecting mice with doxorubicin (DOX), Fang et al. demonstrated that ferroptosis is associated with cardiomyopathy [17]. They also discovered that HO-1 is activated at a higher rate in diseased mice than in control mice. Based on these studies, we suggest that the effect of the Nrf2/HO-1 signaling pathway in cardiovascular diseases may be linked to ferroptosis.

### 2.4. Nrf2/HO-1 Signaling Axis in Pyroptosis

Pyroptosis, characterized by caspase-1 dependence and the release of a large number of pro-inflammatory cytokines, is a newly identified type of programmed cell death. The morphological characteristics, occurrence, and regulatory mechanisms of pyroptosis are different from those of other cell death modes such as apoptosis and necrosis.

The inflammatory response involved in pyroptosis is closely related to the Nrf2/HO-1 signaling axis. Zhao et al. found that downregulation of the Nrf2/HO-1 pathway in neuroblastoma cells activated cell pyroptosis and increased oxidative stress‐induced toxicity [18]. The cyclooxygenase-2 inflammatory signaling cascade pathway, as summarized by Kobayashi et al., confirmed that the downstream product of the cyclooxygenase-2 pathway binds to Keap1, thereby activating Nrf2 and promoting inflammation regression [19]. Zhang et al. recently demonstrated the effective regulation of HO-1 in reducing inflammation and oxidative stress [4, 19]. Furthermore, Chen et al. found that the lipopolysaccharide-induced AMPK/Nrf2/CXC chemokine receptor 2 axis can promote the recruitment of neutrophils to the heart, significantly reducing myocardial dysfunction in mice [20]. Therefore, Nrf2 upregulates neutrophils activity, alleviating lipopolysaccharide-induced cardiac pyroptosis. The evidence implies a protective function of upregulating the Nrf2/HO-1 axis in cardiovascular diseases.

### 2.5. Nrf2/HO-1 Signaling Axis in Autophagy

Autophagy is the process of phagocytosis of cytoplasmic proteins or organelles and their inclusion into vesicles. These vesicles then fuse with lysosomes to form autophagy lysosomes, which is followed by degradation of the contents of the lysosomes for realization of the metabolic needs of the cell and for the renewal of some organelles. It is a dynamically regulated self-digestion process and an endogenous protective mechanism that inhibits myocardial and vascular injury by reducing the release of interstitial vesicles.

There have been multiple reports on the effects of Nrf2-induced autophagy. Inoue et al. found that, under iron deficiency conditions, the body activates Nrf2 expression by regulating the p62/SQSTM1/sequestosome-1 signal during autophagy [21]. Yao et al. demonstrated that activation of the Nrf2‐ARE signaling pathway promotes autophagy in vascular smooth muscle cells and alleviates vascular calcification in high phosphoric conditions [22]. Previous studies have shown that, under oxidative stress, autophagy processes compensate each other to promote cell survival [22]. Recently, it has been found that Nrf2 mRNA levels increased during hepatitis C virus replication, suggesting that the protein kinase RNA-like endoplasmic reticulum kinase axis controls the transcription of antioxidant genes [23].

Based on these reports, we speculate that high levels of cellular stress, especially with the induction of Nrf2, may activate other forms of autophagy to improve cell survival. Now that evidence suggests that Nrf2 signals activate cell survival programs by degrading tumor suppressors and activating oncogenic signals [23], it is reasonable to assume that induction of the Nrf2/HO-1 signaling axis could be a new and efficient clinical treatment method for cardiovascular diseases, especially cardiac tumors and ischemia-reperfusion diseases.

### 2.6. Nrf2/HO-1 Signaling Axis in Programmed Cell Necrosis

Necrosis was recognized as an unregulated form of cell death, but a growing number of studies have shown that necrosis is a highly regulated process. Programmed cell necrosis is a type of necrosis. Adameova et al. found that in the absence of lethal stress or injury, programmed necrosis of cardiomyocytes is a major factor that may induce cardiovascular diseases [24]. Zhu and Sun stated that programmed cell necrosis plays a crucial role not only in heart homeostasis but also in the pathogenesis of cardiovascular diseases [25]. de Souza Prestes et al. demonstrated that patients with cardiovascular diseases have a higher level of methylglyoxal (MG), which can be controlled by the Nrf2/HO-2 signaling axis and converted to D-lactic acid [26]. Moreover, the increase in MG content is closely related to cell necrosis [26]. These data indicate that Nrf2/HO-1 is associated with programmed cell necrosis.

### 2.7. Nrf2/HO-1 Signaling Axis in Alkaliptosis

Recently, new forms of nonapoptotic regulated cell deaths (RCD), such as alkaliptosis, have been studied. Alkaliptosis is a unique pH-dependent form of RCD driven by intracellular alkalinization [27]. Song et al. showed that JTC801 can induce a pH-dependent cell death in human pancreatic cancer cells by activating NF-*κ*B [28]. This form was found to be distinct from ferroptosis, necroptosis, and autophagy, and was defined as alkaliptosis. As we have pointed above, NF-*κ*B is closely related to Nrf2/HO-1 signaling axis. Therefore, we suggest that alkaliptosis and Nrf2/HO-1 signaling axis are related.

Although NF-*κ*B inhibitors can inhibit alkaliptosis [29], no effect of Nrf2 knockout alters JTC801-induced cell death [28]. However, as pathological significance of alkaloptosis and its core effector molecules are still being studied, the specific relationship is not yet clear. In addition, Song et al. are focusing on pancreatic cancer rather than cardiovascular disease, and the study has limitations. Therefore, based on the close relationship between Nrf2/HO-1 and NF-*κ*B, we hypothesize that Nrf2/HO-1 causes cardiovascular diseases through alkalinization.

### 2.8. Nrf2/HO-1 Signaling Axis in Clockophagy

Disruption of circadian rhythms exacerbates disease pathogenesis. Profiling of mice genes showed that 43% of all protein-coding genes display a biological rhythm, mostly in an organ-specific manner [30]. de la Sierra et al. have demonstrated that clockophagy is associated with hypertension [31], which indicates that clockophagy has a link with cardiovascular diseases. Melissa et al. found that the circadian regulation of protein expression plays a significant role in the cellular response to oxidative stress. They also concluded that the different levels of lipid peroxidation and protein oxidation in the day indicate circadian oscillations of oxidative stress responses. This rhythmicity of antioxidant levels can be exploited for a more precise targeting of ROS [32]. Yang et al. indicated that clockophagy is the endogenous oscillating mechanism, as it controls various cellular processes, including iron metabolism, oxidative stress, and cell death [33]. Liu et al. demonstrated that clockophagy, namely, the selective autophagic degradation of the circadian clock regulator aryl hydrocarbon receptor nuclear translocator-like protein/brain and muscle ARNT-like 1 (ARNTL/BMAL1), promotes ferroptosis in vitro and in vivo [34]. In other words, clockophagy-mediated ARNTL degradation promotes lipid peroxidation and subsequent ferroptosis. These studies indicate that an all-new type of selective autophagy can promote ferroptosis via the Egl 9 homolog 2-hypoxia-induced factor 1A (EGLN2-HIF1A) pathway. As ferroptosis can have an effect on cardiovascular diseases, it is closely related to clockophagy; we suggest that there is a relationship between cardiovascular diseases and clockophagy.

## 3. Clinical Research in Pediatric Cardiovascular Diseases

### 3.1. Viral Myocarditis

Viral myocarditis refers to an infectious cardiomyopathy, which can be localized to myocardium or diffuse inflammatory disease caused by viral infection. Viral myocarditis is usually caused by enteroviruses, including Coxsackie virus B (CVB). Patients suffering from viral myocarditis show symptoms such as chest pain, rapid or abnormal heart rhythms, and shortness of breath.

According to Song et al., ulinastatin (UTI) protects against CVB3-induced acute viral myocarditis through Nrf2 activation [35]. Furthermore, Ai et al. revealed that Nrf2 pathway is downregulated because of the upregulation of the 12/15-lipoxygenase (12/15-LO), which can be inhibited by baicalein through reducing inflammatory cytokine production and oxidative stress [35, 36]. Conversely, Wang et al. have demonstrated that the immune and inflammatory processes can also function as a trigger for the activation of Nrf2 [37].

The Nrf2 pathway has not yet been systematically researched in the context of the treatment of viral myocarditis; further research will reveal the effects of this pathway.

### 3.2. Rheumatic Heart Disease (RHD)

Common in remote communities, RHD is a disease involving severe damage to the human heart caused by rheumatic fever. RHD often develops from untreated streptococcal infection, leading to an inflammatory condition.

Similar to RHD, rheumatic arthritis (RA) also results in immune activation, inflammation, and oxidative stress [38]. In response to oxidative stress, the activation of Nrf2/HO-1 exhibits anti-inflammatory and antioxidative effects in animal and human models of RA [39].

Although there are few studies investigating the function of Nrf2 in RHD, with numerous reports regarding Nrf2 functioning in the anti-inflammatory process, we can reasonably speculate that the Nrf2 pathway plays an important role in recovery from RHD.

### 3.3. Cardiac Tumors

Cardiac tumors are primary or secondary tumors that form in the heart. Whether the tumor is benign or malignant, it causes problems because of its location and size, which is large enough to be discovered by echocardiogram. Many patients have no symptoms; however, if blood flow is blocked, it results in shortness of breath.

Nrf2 pathway can be “fine-tuned” by microRNA (miRNA) to regulate antioxidant defense enzymes and counteract oxidative stress [40]. Furthermore, while studying sulforaphane, Bose et al. found that Nrf2 can be activated to protect heart from DOX toxicity, whereas DOX is functioning synergistically with sulforaphane [41]. Therefore, we can hypothesize that Nrf2 has a significant effect on cardiac tumors.

In future, methods for studying miRNAs and cardiac tumors will be developed, which could reveal the potential of the Nrf2 pathway for the treatment of cardiac tumors.

### 3.4. Myocardial Infarction (MI)

MI occurs when the blood flow to the heart is insufficient, and it causes damage to cardiac muscles. MI is often caused by high blood pressure and myocarditis, and results in abnormal heart rate, sudden breathlessness, and extreme fatigue. MI, which is difficult to treat, has gradually become one of the most severe diseases worldwide.

According to Lian et al., in addition to downregulating the expression of several inflammatory factors and activating apoptosis-related proteins, MI model of rats having progressive nephropathy activate the renin-angiotensin-aldosterone system (RAAS), which is simultaneously connected with the defective Nrf2 pathway [42]. Hence, restoration of Nrf2 may have a positive influence on the RAAS. Furthermore, based on the consideration that Nrf2 reduces oxidative stress and that tetrahedral DNA nanostructures (TDNs) are biologically safe, Zhang et al. have discovered that TDNs can activate the Nrf2 pathway to improve the outcome after a myocardial infarction injury, by being transferred into the nucleus and upregulating genes involved in antioxidative mechanisms [43]. Ren et al. also discovered a pathway which cooperates with Nrf2 to maintain sufficient oxygen availability [44].

An increasing amount of research has been published to support the theory that Nrf2 pathway can be used in the recovery of MI. It is believed that a simplified method for utilizing the Nrf2 pathway will soon be applied to clinical settings.

### 3.5. Myocardial Ischemia Reperfusion Injury (MIRI)

MIRI is an adverse cardiovascular condition caused by blood rushing back to the heart after a period of no blood flow. The lack of blood results in a shortage of oxygen and nutrients, which are necessary for heart functioning, whereas the return of blood flow causes inflammation and damage.

In MIRI, Nrf2 downregulates the inflammatory factor myeloperoxidase (MPO) and simultaneously upregulates the levels of antioxidants, such as SOD and glutathione peroxidase (GPx), to counter inflammation and oxidative stress. Jakobs et al. demonstrated that Nrf2 is maintained in the reduced state to increase the expression of antioxidative enzymes by thioredoxin-1 (Trx-1), and in turn, Nrf2 regulates the activation of Trx-1 through two AREs [45]. As demonstrated by Chen et al., Nrf2 is able to not only function after inflammation and oxidative stress but also after reperfusion injury, via activation by trigger factors such as emulsified isoflurane (EI) [46].

Though Nrf2 has a mostly indirect effect on MIRI, its potential for recovery from myocardial damage must not be neglected. When used with other clinical treatments, the Nrf2 pathway can significantly reduce inflammation and oxidative stress, thereby alleviating MIRI. However, the trials are still in the animal testing phase, and more experiments are required to prove Nrf2 as a therapeutic strategy.

### 3.6. Atherosclerosis (AS)

Atherosclerosis is a disease that hardens and narrows the arteries, risking reduced blood flow. AS usually results from damage of the endothelium from high blood pressure, smoking, and high cholesterol. If allowed to worsen, AS can lead to cardiovascular diseases.

Yang et al. demonstrated that the leaf extract of *Nelumbo nucifera* (NLE) possesses anti-atherosclerosis properties. The supplementation of NLE also increases the expression of Nrf2 and its downstream targets [47]. Hence, using NLE to upregulate the function of Nrf2 can be developed into a rational treatment for atherosclerosis. Li et al. put forward a similar method by utilizing bisdemethoxycurcumin (BDMC), extracted from turmeric, to enhance the expression of HO-1 [48]. In Nrf2/HO-1-dependent manner, BDMC provides cardioprotection and can be used in clinical treatments. Girona et al. have found that Nrf2 is activated by palmitate, and may represent a new mechanism of mediating atherosclerosis via saturated fatty acids [49]. AS has been shown to be triggered by multiple mechanisms. However, with the discovery of the Nrf2/HO-1 signaling axis, more targeted treatment for patients is possible.

### 3.7. Arrhythmia

Abnormal conduction by the heart can cause tachycardia, bradycardia, and arrhythmia. Arrhythmias are particularly common in patients with heart disease and often occur during anesthesia, surgery, or after surgery. Stress, smoking, drinking, excessive fatigue, and serious insomnia often result in arrhythmia.

Studies have shown that excessive ROS not only cause arrhythmia but also cause inflammatory body infiltration and activate autoimmunity [50], which may further damage cardiomyocytes. In addition, Dai et al. pointed out that long-term arrhythmia may cause or enhance fibrosis-induced chronic cardiomyopathy [51], which may lead to severe long-term damage in patients.

However, the exploration of the Nrf2/HO-1 axis has revealed a possible solution. Dong et al. showed that when Nrf2 expression is inhibited in H9C2 cells, aldehydes no longer stimulate the production of enough antioxidant enzymes to reverse the oxidative damage, leading to a greatly increased incidence of arrhythmias [52]. On the contrary, Enayati et al. found that *Potentilla reptans* extract directly enhances endogenous antioxidant defense of cardiomyocytes and indirectly activates the NO pathway through the Nrf2/HO-1 signaling axis, thereby relieving arrhythmia and myocardial infarction [53].

The activation of the Nrf2 signaling pathway can inhibit oxidation and reduce the production of free radicals, thereby reducing the oxidative damage to the myocardium. Research so far is restrained to cellular and animal experiments. Hopefully, Nrf2-based therapies will be tested in human clinical trials in the near future.

### 3.8. Hypertensive Heart Disease

Hypertensive heart disease results from the failure to control long-term hypertension, which leads to defects in the heart structure. Some studies show that 70% of heart failures are caused by high blood pressure. At the same time, high blood pressure may cause coronary heart disease, atrial fibrillation, and other cardiac complications, finally resulting in heart failure.

Previous studies have proposed that mitochondrial dysfunction may contribute to the development of cardiovascular diseases, such as hypertensive heart disease [54]. According to our theory summarized above, the Nrf2/HO-1 signaling axis may reverse the effect of mitochondrial apoptosis, thus slowing down the disease process, and could be used as a therapy to alleviate hypertensive heart disease. Furthermore, Wang et al. revealed that rosuvastatin can improve cardiac function and reduce hypertrophy by regulating the interaction between Nrf2 and Smads [55]. Upregulation of the Nrf2/ARE/HO-1 pathway and downregulation of the transforming growth factor 1 (TGF-1)/Smads pathway play important roles in myocardial remodeling.

A recent study by Biernacki et al. found that inhibition of lipolysis by fatty acid amide hydrolase inhibitors can increase enzymatic activity and nonenzymatic antioxidant activity in rats, whereas Nrf2 expression is significantly downregulated [56]. Clinical trials have just started, and the specific treatments are now in the molecular and animal testing phases. Therefore, treatments involving the upregulation of Nrf2 expression will improve hypertensive heart disease by promoting lipolysis in the near future (Table.  1)

## 4. Conclusions

The Nrf2/HO-1 signaling pathway is a multi-organ protection pathway, with an antioxidative function, playing a role in eliminating environmental and endogenous stressors from the body, thus delaying the progress of related diseases. This pathway has become a popular target for research on the occurrence and development of oxidative stress-related diseases. This has a complex regulatory mechanism in oxidative stress-related diseases. It plays a role in reducing mitochondrial damage, and regulating Ca^2+^ influx, programmed cell death, autophagy, and cell pyroptosis and ferroptosis. Latest research also suggests two new processes in which the Nrf2/HO-1 axis is involved: alkaliptosis and clockophagy. Through in-depth research and exploration of the mechanisms, good theoretical support for the therapeutic application of Nrf2/HO-1 in clinical practice can be provided. Hopefully, the diseases mentioned above may well have a new therapy in the future.

## Figures and Tables

**Table 1:** Functional effects of the Nrf2/HO-1 signaling axis in cardiovascular diseases.

Cardiovascular disease	Effects	Reference
Viral myocarditis	Reduces inflammatory cytokine production and oxidative stress	[36]
	Reduces CVB3-induced myocarditis by activating GSK-3*β*	[37]
Rheumatic heart disease (RHD)	Inhibits signal transducer	[39]
Cardiac tumors	Regulates antioxidant defense enzymes and counteracts oxidative stress	[40]
	Reduces toxicity with sulforaphane	[41]
Myocardial infarction (MI)	Involved the renin-angiotensin-aldosterone system	[42]
	Upgrades antioxidant genes	[43]
Myocardial ischemia reperfusion injury (MIRI)	Regulates the activation of thioredoxin-1 (Trx-1) through two antioxidant response elements	[45]
	Regulates ROS levels	[46]
Atherosclerosis (AS)	Strengthens antioxidative potential and alleviates inflammation	[47]
	Enhances the expression of HO-1	[48]
	Mediates atherosclerosis by saturated fatty acids	[49]
Arrhythmia	Produces antioxidant enzymes to reverse oxidative damage	[52]
	Activates the NO pathway	[53]
Hypertensive heart disease	Reverses the mitochondrial apoptosis effect	[54]
	Downregulates TGF-1/Smads in myocardial remodeling	[55]
	Promotes lipolysis enzymatic activity	[56]
